# 

**Published:** 1893-10

**Authors:** 


					﻿Vol. XIII.
OCTOBER, 1893.
No. 10?
THE
JIOMCEOPATHIC pHY^lClAjI,
A Monthly Journal of Homoeopathic Materia Medica
and Clinical Medicine.
"He it a freeman whom truth makes free, and all are slaves beside."
"Seek the Truth: come whence it may, cost what it will."
EDITED AND PUBLISHED BY
WALTER M. JAMES, M. D.
PHILADELPHIA :
No.	BipRTTCTD STRSJBZr.
LONDON’ :
ALFRED HEATH & CO., Sole Agents for Great Britain,
114 Ebury Street, Eaton Square.
Yearly Subscription, $2.50, invariably in advance. Single numbers, 95 els.
Entered at the Philadelphia PeavOffiee aa Second Clue Mall Matter.
				

